# Omnidirectional flat bands in chiral magnonic crystals

**DOI:** 10.1038/s41598-022-20539-3

**Published:** 2022-10-25

**Authors:** J. Flores-Farías, R. A. Gallardo, F. Brevis, Alejandro Roldán-Molina, D. Cortés-Ortuño, P. Landeros

**Affiliations:** 1 Departamento de Física, Universidad Técnica Federico Santa María, Avenida España 1680, Valparaíso, Chile; 2 Universidad de Aysén, Calle Obispo Vielmo 62, Coyhaique, Chile; 3 Paleomagnetic Laboratory Fort Hoofddijk, Department of Earth Sciences, Utrecht University, Budapestlaan 17, 3584 CD Utrecht, The Netherlands

**Keywords:** Magnetic properties and materials, Magnetic devices, Ferromagnetism, Metamaterials, Surfaces, interfaces and thin films

## Abstract

The magnonic band structure of two-dimensional chiral magnonic crystals is theoretically investigated. The proposed metamaterial involves a three-dimensional architecture, where a thin ferromagnetic layer is in contact with a two-dimensional periodic array of heavy-metal square islands. When these two materials are in contact, an anti-symmetric exchange coupling known as the Dzyaloshinskii–Moriya interaction (DMI) arises, which generates nonreciprocal spin waves and chiral magnetic order. The Landau–Lifshitz equation and the plane-wave method are employed to study the dynamic magnetic behavior. A systematic variation of geometric parameters, the DMI constant, and the filling fraction allows the examination of spin-wave propagation features, such as the spatial profiles of the dynamic magnetization, the isofrequency contours, and group velocities. In this study, it is found that omnidirectional flat magnonic bands are induced by a sufficiently strong Dzyaloshinskii–Moriya interaction underneath the heavy-metal islands, where the spin excitations are active. The theoretical results were substantiated by micromagnetic simulations. These findings are relevant for envisioning applications associated with spin-wave-based logic devices, where the nonreciprocity and channeling of the spin waves are of fundamental and practical scientific interest.

## Introduction

Magnonic crystals (MCs) are magnetic materials fabricated in the laboratory with a repeated spatial distribution that creates periodic magnetic properties^1–5^. They are typically prepared in several forms, either from a thin film with regular features, as antidots lattices^6–10^ or surface-modulated MCs^11–13^, or by alternating two different ferromagnetic materials^8,14,15^, or by a periodic array of isolated magnetic nanostructures^7,16^. The main objective of creating and studying magnetic metamaterials is to be able to modify and control the propagation of spin waves^17–21^. Spin waves (SWs) are collective excitations in magnetic materials, which carry information across material regions and are suitable for communication technologies^22,23^. Therefore, it is essential to know the band structure of a magnonic crystal since vital information about the SW propagation can be inferred^8^. MCs can have frequency modes that behave reciprocally concerning the inversion of the wave vector. Nevertheless, under given conditions, SWs exhibit nonreciprocal propagation^24^, which refers to the case where the properties of waves (amplitude, phase, and frequency) change by reversing the direction of propagation^5,25^. Although current research on particular combinations of magnetic materials has proven very useful, metamaterials with one-dimensional chiral periodic features have been only recently explored^26–30^. The tunable properties of magnonic band structures make spin-wave technologies more advantageous than photonic and electronic devices^5^. Indeed, magnonic crystals can be easily controlled in the frequency domain by changing the magnitude and direction of an applied magnetic field or by incorporating anisotropies at the surfaces^24^. Another important property of a magnonic device is the geometry of the system which influences the magnetic energy contributions. In particular, the magneto-dipolar interaction is capable of inducing chiral properties^31–34^. A further ingredient that also adds chiral features is the Dzyaloshinskii–Moriya interaction (DMI)^35–37^, an antisymmetric exchange interaction that causes chiral magnetic order^38–40^, and breaks the symmetry of the spin waves^41–45^. This interaction arises in bulk form in non-centrosymmetric crystals^45–47^ and interfacial systems such as ultrathin magnetic films in contact with a heavy metal (HM) layer with strong spin-orbit coupling^48–50^.

**Figure 1:**
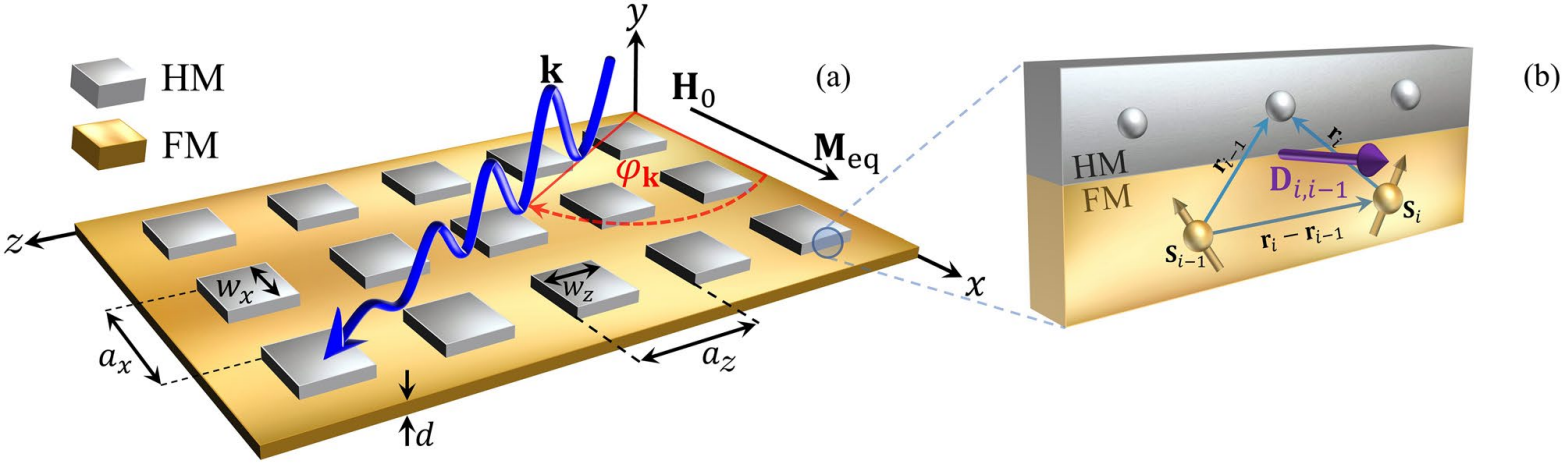
(**a**) Illustration of a ferromagnetic ultrathin film of thickness $d=3$ nm in contact with a periodic array of heavy-metal islands, which induce an interfacial Dzyaloshinskii–Moriya interaction only below the HM. In this 2D chiral magnonic crystal, the spin waves propagate with a wave vector $\mathbf{k}$ in the *x-z* plane with a given angle $\varphi _\mathbf{k}$. The equilibrium magnetization points along *x* due to an external magnetic field applied in the same direction. (**b**) The scheme depicts the notation used to describe the interfacial Dzyaloshinskii–Moriya interaction, where two atomic spins interact through a third Pt atom (gray sphere) at position ${\mathbf {r}}_{i}$ concerning spin *i* and the corresponding DM vector between them.

In magnonic crystals, spin waves are observed in bands of allowed magnon frequencies separated by forbidden bands known as bandgaps (BGs). The magnonic band structure depends on multiple factors, such as the intrinsic material parameters and the geometric design of the magnonic crystal. External conditions, such as the magnitude and direction of both the external magnetic field ${\mathbf {H}}_0$ and the wave vector ${\mathbf {k}}$ provided by the excitation source, also influence the magnonic band structure. A relevant aspect is the nature of the periodic magnetic field within the MC. For instance, in antidot lattices or surface-modulated MCs, the magneto-dipolar contribution produces a periodic magnetic field. On the other hand, it has been shown that dispersionless modes can appear in the spectrum of different forms of MCs, as arrays of coupled ferromagnetic wires^51,52^, bicomponent MCs^53,54^, surface-modulated magnonic crystals^12^, as well as MCs with defects^55^, or magnonic superlattices^56^. Magnonic flat bands have been reported in several types of crystalline spin architectures, including spin-ice compounds^57,58^, honeycomb ferromagnets^59,60^, Kagome-lattice antiferromagnets^61,62^ and ferromagnets^63,64^, Gadolinium Gallium Garnet^65^, and helimagnets hosting periodic magnetic textures^47,66,67^. When a band becomes flat the group velocity is considerably reduced and the associated quasiparticle loses its kinetic energy, allowing the emergence of strongly interacting phases of matter^64,68,69^. In this context, the experimental observation of superconductivity in twisted bilayer graphene^70–72^ is based on the electronic excitations in a flat band^73–75^.

The periodic antisymmetric exchange is a key factor in the development of chiral magnonic crystals, which offers prospects to control the band structure of the spin waves^28,29^. The presence of the DMI as the physical source of the periodic magnetic field causes two physical effects in one-dimensional MCS. First, magnonic Bragg reflections do not balance at the Brillouin zone edges because of the SW nonreciprocity, leading to indirect bandgaps^26,28^. Second, for a strong enough DMI coupling, flat bands are predicted^28^, and further measured^76^, together with a nontrivial SW evolution. This paper is focused on a ferromagnetic ultrathin film covered with two-dimensional periodic arrays of heavy-metal islands (see Fig. 1), where a 2D periodic interfacial DMI is induced. The analysis of the band structure, group velocities, SW localization, and the isofrequency curves of the magnonic crystal suggest the existence of omnidirectional flat bands, where spin waves present almost zero group velocity in all directions. Micromagnetic simulations are employed to confirm the validity of our calculations.

## Theoretical description

### Equation of motion

This section theoretically studies the spin-wave spectrum in two-dimensional chiral magnonic crystals (see Fig. 1a) using the Landau–Lifshitz equation of motion, which describes the temporal evolution of magnetization vector ${\mathbf {M}}\left( {\mathbf {r}},t\right)$:

1 $$\begin{aligned} \frac{\partial {{\mathbf {M}}\left( {\mathbf {r}},t\right) }}{\partial {t}} = -\gamma \mu _{0} {\mathbf {M}}\left( {\mathbf {r}},t\right) \times {\mathbf {H}}^{{\mathrm{{eff}}}}\left( {\mathbf {r}},t\right) . \end{aligned}$$

Here, $\gamma$ is the absolute value of the gyromagnetic ratio, $\mu _{0}$ the permeability of vacuum, ${\mathbf {r}}$ the position vector, *t* the time, and ${\mathbf {H}}^{{\mathrm{{eff}}}}\left( {\mathbf {r}},t\right)$ denotes the effective magnetic field. In the case of a small disturbances, the magnetization vector can be written as ${\mathbf {M}}\left( {\mathbf {r}},t\right) =M_{{\mathrm{eq}}}({\mathbf {r}})\hat{x}+{\mathbf {m}}\left( {\mathbf {r}},t\right)$, where $M_{{\mathrm{eq}}} \gg \vert {\mathbf {m}}\left( {\mathbf {r}},t\right) \vert$. Here, the equilibrium magnetization $M_{{\mathrm{eq}}}({\mathbf {r}})$ corresponds to the static component, and ${\mathbf {m}}\left( {\mathbf {r}},t\right)$ is the dynamic term that oscillate in the *y-z* plane. Similarly, ${\mathbf {H}}^{{\mathrm{{eff}}}}\left( {\mathbf {r}},t\right) = {\mathbf {H}}^{{\mathrm{{eff}}}}_{{\mathrm{{0}}}}\left( {\mathbf {r}},t\right) +{\mathbf {h}}^{{\mathrm{{eff}}}}\left( {\mathbf {r}},t\right)$, where ${\mathbf {h}}^{{\mathrm{{eff}}}}\left( {\mathbf {r}},t\right)$ is linear with the dynamic magnetization ${\mathbf {m}}\left( {\mathbf {r}},t\right)$. By assuming a harmonic time dependence, ${\mathbf {m}}\left( {\mathbf {r}},t\right) ={\mathbf {m}}\left( {\mathbf {r}}\right) e^{-i\,\omega \,t}$, with $\omega =2\pi f$, the LL equation reduces to two coupled equations,

2 $$\begin{aligned}&i \frac{\omega }{\gamma \mu _{0}} m_{y}({\mathbf {r}})=M_{{\mathrm{eq}}}({\mathbf {r}})h^{{\mathrm{{eff}}}}_{z} ({\mathbf {r}})-H^{{\mathrm{{eff}}}}_{0,x}({\mathbf {r}})m_{z}({\mathbf {r}}) , \end{aligned}$$

3 $$\begin{aligned}&i \frac{\omega }{\gamma \mu _{0}}m_{z}({\mathbf {r}})=-M_{{\mathrm{eq}}}({\mathbf {r}})h^{{\mathrm{{eff}}}}_{y}({\mathbf {r}})-H^{{\mathrm{{eff}}}}_{0,x}({\mathbf {r}})m_{y}({\mathbf {r}}) . \end{aligned}$$

The effective field can be written as ${\textbf {H}}^{\mathrm{{eff}}}\left( {\textbf {r}},t\right) ={\textbf {H}}_{0}\left( {\textbf {r}},t\right) +{\textbf {H}}^{\mathrm{ex}}\left( {\textbf {r}},t\right) + {\textbf {H}}^{\mathrm{dip}}\left( {\textbf {r}},t\right) +{\textbf {H}}^{\mathrm{DM} }\left( {\textbf {r}},t\right)$, where the terms at the right are associated with the Zeeman, exchange, dipolar, and interfacial DM interactions, respectively. The plane wave method (PWM) is used to examine the periodic properties of the magnetic structure, which is commonly used to describe photonic, phononic, plasmonic, and magnonic crystals^28,77^. It is a spectral resolution strategy in which the LL equation is transformed into an eigenproblem, which can be solved numerically. According to Bloch’s theorem, the dynamic components of the magnetization vector in a periodic potential are written as ${\mathbf {m}}\left( {\mathbf {r}}\right) =\sum _{{\mathbf {G}}}{\mathbf {m}}_{{\mathbf {G}}}e^{i\left( {\mathbf {G}}+{\mathbf {k}}\right) \cdot {\mathbf {r}}}$. In a bidimensional MC, ${\mathbf {G}}=\left( {2\pi }/{a_{x}}\right) q\text { }\hat{x}+\left( {2\pi }/{a_{z}}\right) p\text { }\hat{z}$ denotes a reciprocal lattice vector of the periodic structure, where *q* and *p* are integers, and $a_{\eta }$ represents the lattice parameter along the $\eta$-axis, while ${\mathbf {k}}$ is the in-plane wave vector. According to this, Eq. (1) becomes $i\frac{\omega }{\gamma \mu _{0}}{\mathbf {m}}_{{\mathbf {G}}}=\tilde{{\mathbf {A}}}{\mathbf {m}}_{{\mathbf {G}}}$, where ${\mathbf {m}}^{{\mathbf {T}}}_{{\mathbf {G}}}=\left[ m_{z}\left( G_{1}\right) \ldots m_{z}\left( G_{N}\right) ,m_{y}\left( G_{1}\right) \ldots m_{y}\left( G_{N}\right) \right]$ are the eigenvectors associated with the dynamical magnetization, and $\tilde{{\mathbf {A}}}$ is the dynamic matrix that contains information about the effective fields. To calculate the eigenvalues and find the eigenfrequencies *f* of the system, the matrix

4 $$\begin{aligned} \tilde{{\mathbf {A}}}= \begin{pmatrix} {\mathrm{A}}^{zz} &{}\quad {\mathrm{A}}^{zy}\\ {\mathrm{A}}^{yz} &{}\quad {\mathrm{A}}^{yy} \end{pmatrix} , \end{aligned}$$

must be diagonalized. The matrix elements are obtained from the effective fields and derived in the following sections.

### Effective fields

The field of the exchange interaction is given by ${\textbf {H}}^{\mathrm{ex}}\left( {\textbf {r}},t\right) =\left[ {\nabla }\cdot \left( \lambda _{\mathrm{ex}}^{2}{\nabla }\right) \right] {\textbf {M}}\left( {\textbf {r}},t\right)$ with $\lambda _{\mathrm{ex}} =\sqrt{2A/(\mu _{0}M_{\mathrm{{s}}}^{\mathrm{{2}}})}$ being the exchange length, $M_{s }$ the saturation magnetization, and *A* the exchange constant. Thus, the exchange field components are

5 $$\begin{aligned} h^{\mathrm{ex}}_{y,z}\left( {\textbf {r}}\right) {=}-\sum _{{\textbf {G}}}  \left( {\textbf {G}}+{\textbf {k}}\right) \lambda ^{2} _{\mathrm{ex}}m_{y,z}\left( {\textbf {G}}\right) e^{\mathrm{i}\left( {\textbf {G}}+{\textbf {k}}\right) \cdot {\textbf {r}}}. \end{aligned}$$

The dipolar field writes as ${\textbf {H}}^{\mathrm{dip}}\left( {\textbf {r}},t\right) =-\nabla U\left( {\textbf {r}},t\right)$, where $U\left( {\textbf {r}},t\right)= -\frac{1}{4\pi } \int _{\nu } \frac{\nabla \cdot {\textbf {m}}\left( {\textbf {r}}',t\right) }{\mid {\textbf {r}}-{\textbf {r}}'\mid }d{\nu }'+ \frac{1}{4\pi } \int _{s} \frac{\hat{{\textbf {n}} }' \cdot {\textbf {M}}\left( {\textbf {r}}',t\right) }{\mid {\textbf {r}}-{\textbf {r}}'\mid }\,d{s}'$ is the magnetostatic potential. Here $\hat{{\textbf {n}} }$ is a unitary vector normal to the plane (parallel to $\hat{y}$). A sufficiently strong external field ${\mathbf {H}}_{0}$ is applied in the *x*-direction in order to saturate the magnetization (see Fig. 1a). The effective dipolar fields are

6 $$\begin{aligned} h^{\mathrm{dip}}_{y}\left( {\textbf {r}}\right)= & {} \sum _{{\textbf {G}}}m_{y}\left( {\textbf {G}}\right) e^{i\left( {\textbf {G}}+{\textbf {k}}\right) \cdot {\textbf {r}}}e^{-\mid {\textbf {G}}+{\textbf {k}}\mid \frac{d}{2}} , \end{aligned}$$

7 $$\begin{aligned} h^{\mathrm{dip}}_{z}\left( {\textbf {r}}\right)= & {} -\sum _{{\textbf {G}}}\frac{\left[ \left( {\mathbf {G}}+{\mathbf {k}}\right) \cdot \hat{z}\right] ^{2}}{\left| {\mathbf {G}}+{\mathbf {k}}\right| ^{2}}m_{z}\left( {\textbf {G}}\right) e^{i\left( {\textbf {G}}+{\textbf {k}}\right) \cdot {\textbf {r}}} \left( 1-e^{-\mid {\textbf {G}}+{\textbf {k}}\mid \frac{d}{2}}\right). \end{aligned}$$

To calculate the DM field, ${\textbf {H}}^{\rm DM }\left( {\textbf {r}},t\right)$, the theory described in Ref.^28^ (for a 1D periodic DMI) is extended to the two-dimensional case. The DM Hamiltonian is described as ${\mathscr {H}}^{\mathrm{{DM}}}=\sum _{i}{\mathbf {D}}_{i-1,i}\cdot \left( {\mathbf {S}}_{i-1}\times {\mathbf {S}}_{i}\right)$, where ${\mathbf {D}}_{i-1,i}=-{\mathbf {D}}_{i,i+1}$ is the DM vector between sites $i-1$ and *i*. This vector related to the coupling between two neighboring FM atomic spins, ${\mathbf {S}}_{i-1}$ and ${\mathbf {S}}_{i}$, with a third nonmagnetic HM site, as illustrated in Fig. 1b. By using vectorial identities, the DM Hamiltonian associated with site *i* becomes ${\mathscr {H}}^{\mathrm{{\,DM}}}=-\sum _{i}{\mathbf {S}}_{i}\cdot {\mathbf {h}}_{i}^{\mathrm{{\,DM}}}$, where ${\mathbf {h}}_{i}^{\mathrm{{DM}}}=-{\mathbf {D}}_{i-1,i}\times {\mathbf {S}}_{i-1} +{\mathbf {D}}_{i,i+1}\times {\mathbf {S}}_{i+1}$. In the case of a two-dimensional periodic DMI, the spin vectors are expanded in the *x-z* plane as ${\mathbf {S}}_{i \pm 1}\simeq {\mathbf {S}}_{i}\pm {\partial _{z} {\mathbf {S}}_{i}}\delta {z}\pm {\partial _{x} {\mathbf {S}}_{i}}\delta {x}$, with the same expansion for the DM vector, ${\mathbf {D}}_{i,i+1}\simeq {\mathbf {D}}_{i-1,i}+\hat{z}\,{\partial _{x}\,D_{i-1,i}}\delta {x}+\hat{x}{\partial _{z} D_{i-1,i}}\delta {z}.$ When the wave propagation is along *z*, the DM vector between spins at *i* and $i+z$ is ${\mathbf {D}}_{i,i+z}=D_{x}\hat{x}$. In the same way, when waves propagate along *x*, the DM vector between spins at *i* and $i+x$ is ${\mathbf {D}}_{i,i+x}=-D_{z}\hat{z}$, since ${\mathbf {D}}_{i-1,i}\perp \left( {\mathbf {r}}_{i}-{\mathbf {r}}_{i-1}\right)$, which must be fulfilled for all propagation directions. Thus, the DM field at site *i* is ${\mathbf {h}}_{i}^{\mathrm{{DM}}} =\left( -2{\mathbf {D}}_{i-1,i}\times {\partial _{x}{\mathbf {S}}_{i}}-{\partial _{x} {\mathbf {D}}_{i-1,i}}\times {\mathbf {S}}_{i}\right) \delta {x}+ \left( -2{\mathbf {D}}_{i-1,i}\times {\partial _{z}{\mathbf {S}}_{i}}-{\partial _{z}{\mathbf {D}}_{i-1,i}}\times {\mathbf {S}}_{i}\right) \delta {z}.$ In the continuous approach, and using $\vert D_x\vert =\vert D_z\vert$, the DM effective field components are

8 $$\begin{aligned} h^{\mathrm{DM}}_{y}\left( {\textbf {r}}\right)= & {} -2i\sum _{{\textbf {G}},{\textbf {G}}'}\frac{D\left( {\textbf {G}}'\right) m_{z}\left( {\textbf {G}}\right) }{\mu _{0}\,M^2_{s}} e^{i\left( {\textbf {G}}+{\textbf {G}}'+{\textbf {k}}\right) \cdot {\textbf {r}}} \left[ {\textbf {G}}+{\textbf {k}}+\frac{{\textbf {G}}'}{2}\right] \cdot \hat{z} \end{aligned}$$

9 $$\begin{aligned} h^{\mathrm{DM}}_{z}\left( {\textbf {r}}\right)= & {} -2i \sum _{{\textbf {G}},{\textbf {G}}'} \frac{D\left( {\textbf {G}}'\right) m_{y}\left( {\textbf {G}}\right) }{\mu _{0}\,M^2_{s}} e^{i\left( {\textbf {G}}+{\textbf {G}}'+{\textbf {k}}\right) \cdot {\textbf {r}}}\left[ {\textbf {G}} +{\textbf {k}}+\frac{{\textbf {G}}'}{2}\right] \cdot \hat{z}, \end{aligned}$$

where $D({\mathbf {G}})$ is the DM constant that is periodic and thus relies on the lattice vectors. Taking into account all field contributions, including the external field ${\mathbf {H}}_0=H_0 \hat{x}$, the matrix elements depicted in Eq. (4) are

10 $$\begin{aligned} {\mathrm{A}}_{{\mathbf {G}},{\mathbf {G'}}}^{zz}= & {} {\mathrm{A}}_{{\mathbf {G}},{\mathbf {G'}}}^{yy}=-2i\frac{D \left( {\mathbf {G}}-{\mathbf {G'}}\right) }{\mu _{0}M_{s }}\left[ \frac{{\mathbf {G}}+{\mathbf {G'}}}{2} +{\mathbf {k}}\right] \cdot \hat{z}, \end{aligned}$$

11 $$\begin{aligned} {\mathrm{A}}_{{\mathbf {G}},{\mathbf {G'}}}^{zy}= & {} -\lambda _{\mathrm{ex}}^{2}M_{s }\delta _{{\mathbf {G}}, {\mathbf {G'}}}\left( {\mathbf {G'}}+{\mathbf {k}}\right) ^{2}-\frac{\left[ \left( {\mathbf {G'}}+{\mathbf {k}}\right) \cdot \hat{z}\right] ^{2}}{\left| {\mathbf {G'}}+{\mathbf {k}}\right| ^{2}}M_{s }\delta _{{\mathbf {G}}, {\mathbf {G'}}}\left[ 1-e^{-\left| {\mathbf {G'}}+{\mathbf {k}}\right| \frac{d}{2}}\right] -H_{0}\delta _{{\mathbf {G}},{\mathbf {G'}}}, \end{aligned}$$

12 $$\begin{aligned} {\mathrm{A}}_{{\mathbf {G}},{\mathbf {G'}}}^{yz}= & {} \lambda _{\mathrm{ex}}^{2}M_{s }\delta _{{\mathbf {G}}, {\mathbf {G'}}}\left( {\mathbf {G'}}+{\mathbf {k}}\right) ^{2}+M_{s }\delta _{{\mathbf {G}},{\mathbf {G'}}} e^{-\left| {\mathbf {G'}}+{\mathbf {k}}\right| \frac{d}{2}}+H_{0}\delta _{{\mathbf {G}},{\mathbf {G'}}}. \end{aligned}$$

These matrix elements allow calculating the SW spectra for the two-dimensional magnonic crystal system. Eigenvalues of Eq. 4 are associated with the frequencies, while the eigenvectors allow obtaining the corresponding dynamic magnetization components.

## Micromagnetic simulations

Micromagnetic simulations, based on the GPU-accelerated code MuMax3^78^, are performed to validate the theoretical model. For this purpose, it is considered an ultrathin magnetic stripe with dimensions of $10\; {\upmu {\mathrm{m}}} \times 300\;{\mathrm {nm}}\times 3\;{\mathrm {nm}}$ along the (*z*, *x*, *y*) components respectively, and discretized into $2^{12} \times 2^{7} \times 1$ cells. Then, periodic boundary conditions are used along both the *x* and *z* directions to simulate an extended film. Periodic DMI was implemented as follows: MuMax3 allows defining regions with particular magnetic properties but interacting with each other. In this case, two periodic regions were defined: one in which the DM constant is zero and the other in which it has a finite value *D*. For the Damon-Eshbach (DE) configuration (i.e., wave vector along the *z*-direction), the system was initialized with the magnetization along the *x*-direction, parallel to the applied magnetic field of $250\; {\mathrm{mT}}$. In order to generate SWs, the since pulse ${\mathbf {h}}= h_{0} {\mathrm{sinc}}(2\pi f_{\mathrm{c}}t)\hat{z}$ was applied at the center of the film over a width of 40 nm in $\hat{z}$ with $h_{0} = 25$ mT, and a cut-off frequency of $f_{\mathrm{c}} = 50$ GHz. The system evolved for 25 ns, and the magnetization was stored every 0.5 ps. The same material parameters has been used for micromagnetic simulations and PWM calculations. The dispersion relation was obtained by calculating the two-dimensional fast Fourier transform in time and space, of the stored data. For the backward volume (BV) configuration, the procedure was the same as before, the magnetization was initialized and saturated along the *x*-direction, but in this case, the applied pulse was in the form of ${\mathbf {h}}= h_{0}{\mathrm{sinc}}(2\pi f_{\mathrm{c}}t)\hat{x}$.

## Results and discussion

For the calculations, a permalloy (Ni$_{80}$Fe$_{20}$)^79,80^ film with thickness $d=3$ nm is chosen, so that the saturation magnetization is $M_{\mathrm{{s}}}=658$ kA/m^81^, the exchange length is $\lambda _{\mathrm{ex}}=6.39$ nm^82^ and the exchange constant is $A=11.1$ pJ/m$^2$ (Ref.^82^ uses an exchange constant $A=11$ pJ/m$^2$ and gives an exchange length $\lambda _{\mathrm{ex}}=6.36$ nm). The gyromagnetic ratio $\gamma =175.866$ GHz/T. In all calculations, a bias field $\mu _{0}H=250$ mT is applied along *x*, while the SWs propagate in the *x-z* plane. It is worth mentioning that at small fields there is a formation of magnetic textures as *D* increases^47^. Therefore, we have chosen a strong magnitude of the external magnetic field to keep the saturated state stable.

**Figure 2:**
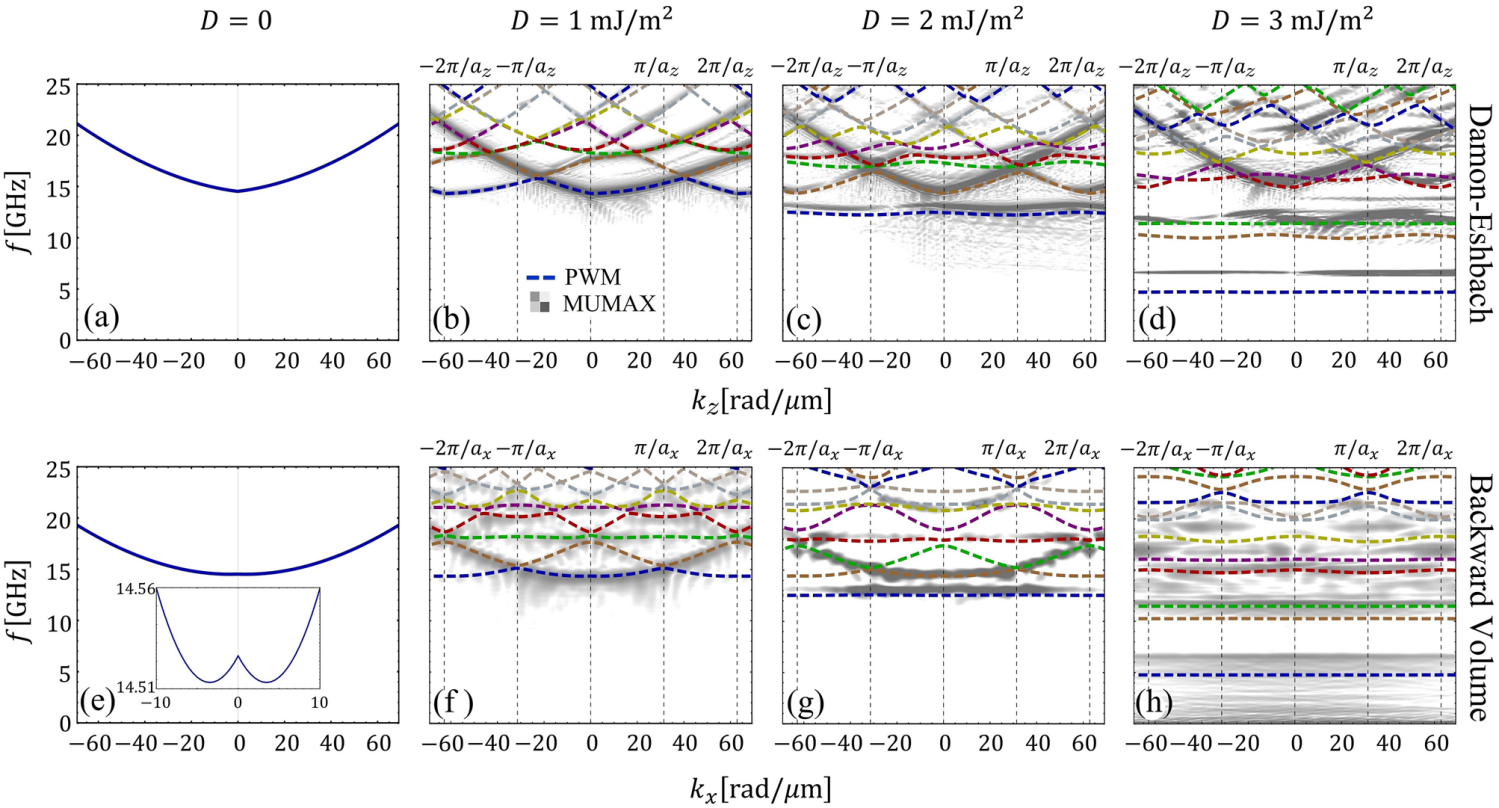
Magnonic band structure for an ultrathin film in contact with an array of square heavy-metal islands with period $a_{z}=a_{x}=100$ nm and $w_{z}=w_{x}=50$ nm. (**a**–**d**) Shows the Damon–Eshbach case and (**e**–**h**) the backward volume configuration, for $D=0,1,2$ and 3 mJ/m$^2$. The inset in (**e**) depicts the typical SW dispersion of the backward volume geometry, wherein the mode exhibits two minima at finite wave vectors. The dashed lines correspond to the calculations and the gray code to the micromagnetic simulation.

According to Fig. 1, $\varphi _\mathbf{k}=90^{\circ }$ for spin waves propagating in Damon–Eshbach configuration, that is ${\mathbf {M}}_{\mathrm{{eq}}}\perp {\mathbf {k}}$. For the spin waves propagating in the backward-volume configuration, $\varphi _\mathbf{k}=0^{\circ }$ so that ${\mathbf {M}}_{\mathrm{{eq}}}\parallel {\mathbf {k}}$. Although the DE and BV configurations are typical experimental setups, the band structure, group velocity, isofrequency contours, and dynamic profiles are calculated for any value of the SW angle $\varphi _\mathbf{k}$. It is worth mentioning that DE and BV configurations are different in terms of the SW propagations because the effects of the DMI are distinct for both cases.

The spin-wave dispersion for squared heavy-metal islands is shown in Fig. 2 for $a_{z}=a_{x}=100$ nm, and heavy-metal widths $w_{z}=w_{x}=50$ nm, while the DMI strength takes values of $D=0$, 1, 2 and 3 mJ/m$^{2}$, which are in concordance with values measured experimentally^29,83^. The standard antisymmetric character of the interfacial DMI can be observed for the Damon-Eshbach configuration. Bandgaps are observed for both DE and BV configurations. In the DE configuration, Fig. 2b–d show that as *D* increases, the effects of nonreciprocity caused by the periodic DMI are more pronounced. Indirect bandgaps for DE modes are observed, where the minimum of the second band and the maximum of the first band occurs at different wave vectors. In Fig. 2c, indirect gaps appear even for small *D* because of the SW nonreciprocity, which shifts the minimum and maximum of the bands from the Brillouin zones (vertical lines) distinctively. This behavior has been reported previously in a one-dimensional chiral MC^28^. In the case of the BV configuration (Fig. 2e–h), a symmetric character of the SWs is observed since the DMI contribution and the frequency nonreciprocity vanishes for $\varphi _\mathbf{k}=0$^43^. Overall, we observe a general behavior for both configurations DE and BV, wherein under the increase of the constant *D*, the bands move to lower frequencies, and at the same time, the low-frequency bands are flattened. A reasonable agreement is observed between the calculations and the micromagnetic simulations, where both methods show the flattening of the low-frequency bands and the indirect character of the bandgaps.

**Figure 3:**
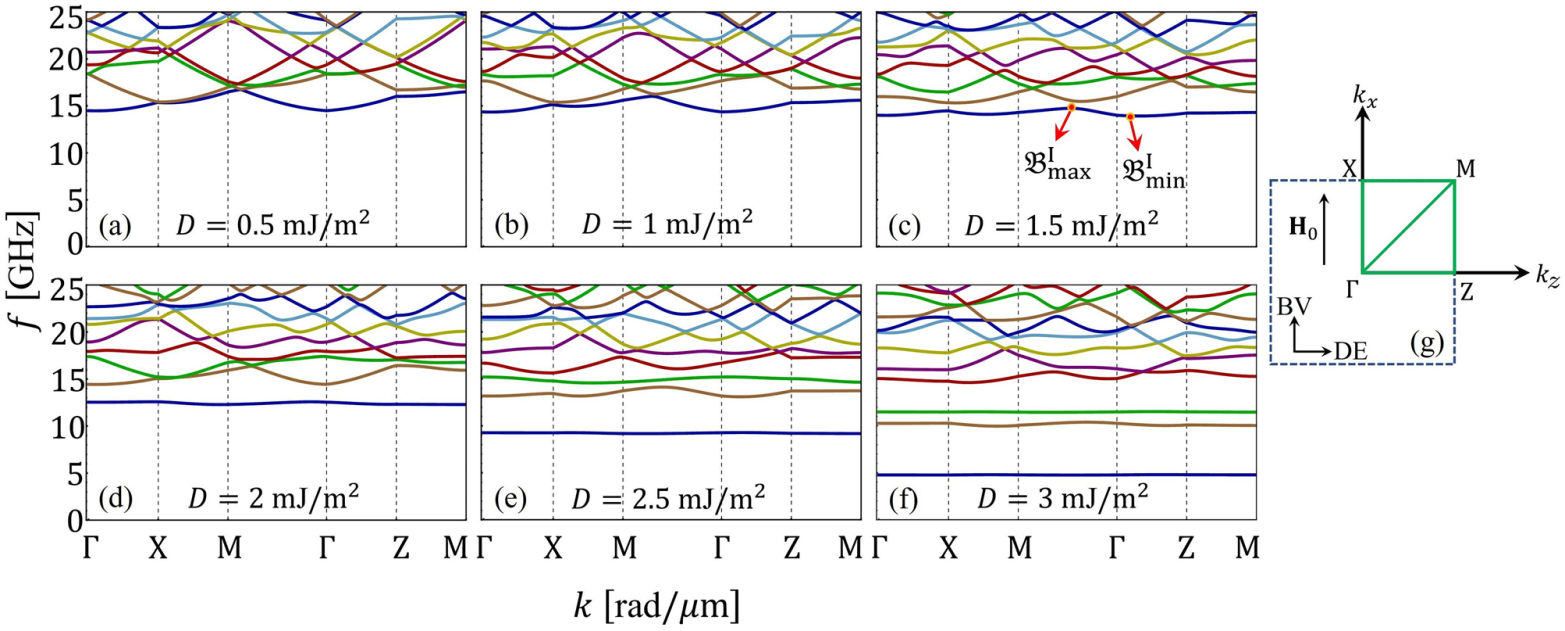
(**a**–**f**) Show the band structures of a two-dimensional chiral magnonic crystal on the reduced Brillouin zone, where the path followed by the wave vector is shown in (**g**). Heavy-metal islands are considered for inducing a periodic Dzyaloshinskii–Moriya coupling, where $a_{z}=a_{x}=100$ nm and $w_{z}=w_{x}=50$ nm. The states ${\mathfrak {B}}^{n}_{\mathrm{max}}$ and ${\mathfrak {B}}^{n}_{\mathrm{min}}$ are defined in (**c**), which describe the maximum and minimum of the band *n*, respectively.

The band structure in the reduced Brillouin zone is studied as a function of *D*, allowing us to explore the SW propagation in all in-plane directions. It is observed from Fig. 3 that, as the DM strength increases, the bandgap widths become more significant, while the low-frequency bands become flat. The essential point is that the dispersionless bands are a global feature of the system, in such a way that in all propagation directions, there are flat bands for a large value of *D*, so that the low-frequency modes have an omnidirectional flat character. To systematically analyze the flattening of the bands, the points ${\mathfrak {B}}^{n}_{\mathrm{max}}$ and ${\mathfrak {B}}^{n}_{\mathrm{min}}$ are defined (see Fig. 3c), where *n* is the number of a specific band ($n={\mathrm {I,II,III}}\,\,{\mathrm {and}}\,\, {\mathrm {IV}}$, being I the lowest frequency band).

**Figure 4:**
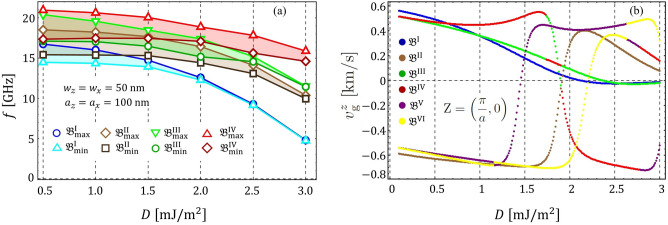
(**a**) Minimum and maximum values of the magnonic bands (${\mathfrak {B}}^{n}_{\mathrm{min}}$ and ${\mathfrak {B}}^{n}_{\mathrm{max}}$) as a function of the DM constant *D*. (**b**) *z*-direction component of the group velocity for the first 6 low-frequency bands at the Z point of the reduced Brillouin zone (DE configuration).

**Figure 5:**
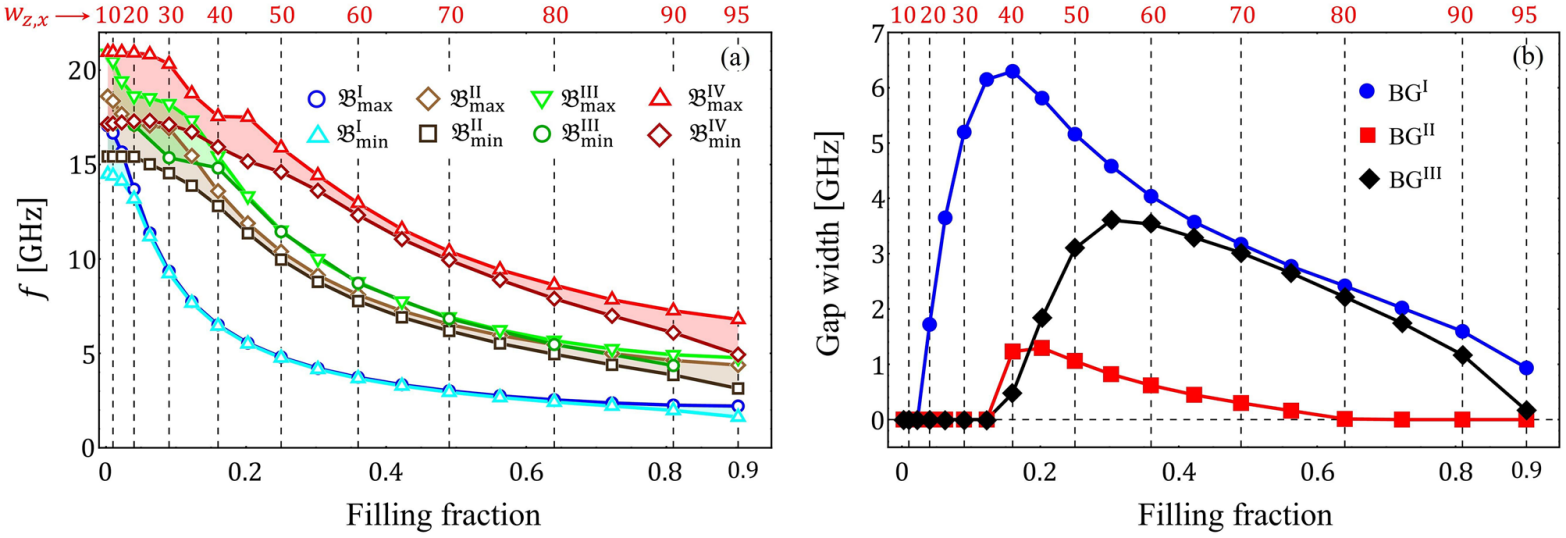
(**a**) Minimum and maximum values for the first four low-frequency bands as a function of the filling fraction. When the difference between the maximum and the minimum becomes negligible, tha band becomes nearly flat. The case $w_{z}=w_{x}=50$ nm, $a_{\,z}=a_{\,x}=100$ nm and $D=3$ mJ/m$^{2}$ is considered. (**b**) Bandgap widths of the first three bandgaps, calculated in the reduced Brillouin zone, are depicted as a function of the filling fraction.

Figure 4 illustrates the points ${\mathfrak {B}}^{n}_{\mathrm{max}}$ and ${\mathfrak {B}}^{n}_{\mathrm{min}}$ as a function of *D* for $a_{z}=a_{x}=100$ nm and $w_{z}=w_{x}=50$ nm. The results consider the first four low-frequency bands. By analyzing the difference between the points ${\mathfrak {B}}^{n}_{\mathrm{max}}$ and ${\mathfrak {B}}^{n}_{\mathrm{min}}$, we can explicitly see the formation of flat bands. For instance, the first band becomes flat at $D\approx 2$ mJ/m$^{2}$, while the high-frequency modes require a significant value of *D* (more than 3 mJ/m$^{2}$) to reach the dispersionless character. In what follows, if the difference between the maximum ${\mathfrak {B}}^{n}_{\mathrm{max}}$ and the minimum ${\mathfrak {B}}^{n}_{\mathrm{min}}$ of the *n*-th band is less or equal to 0.1 GHz (${\mathfrak {B}}^{n}_{\mathrm{max}}-{\mathfrak {B}}^{n}_{\mathrm{min}}\le 0.1$ GHz) the band will be referred as a flat band. The formation of flat bands is linked to the group velocity, which can be calculated from its definition ${\mathbf {v}}_{\mathrm{{g}}} =\nabla _{\mathbf {k}}\omega ({\mathbf {k}})$. Thus, if the slope (in the *f* vs. *k* plot) is large, then the group velocity’s magnitude will also be large, while its direction is given by the sign on the slope. As an example, Fig. 4b shows the *z*-direction of the group velocity, $v^{z}_{\mathrm{g}}$, of the first four low-frequency bands at point Z, ($\pi /a_z,0$), of the reduced Brillouin zone.

**Figure 6:**
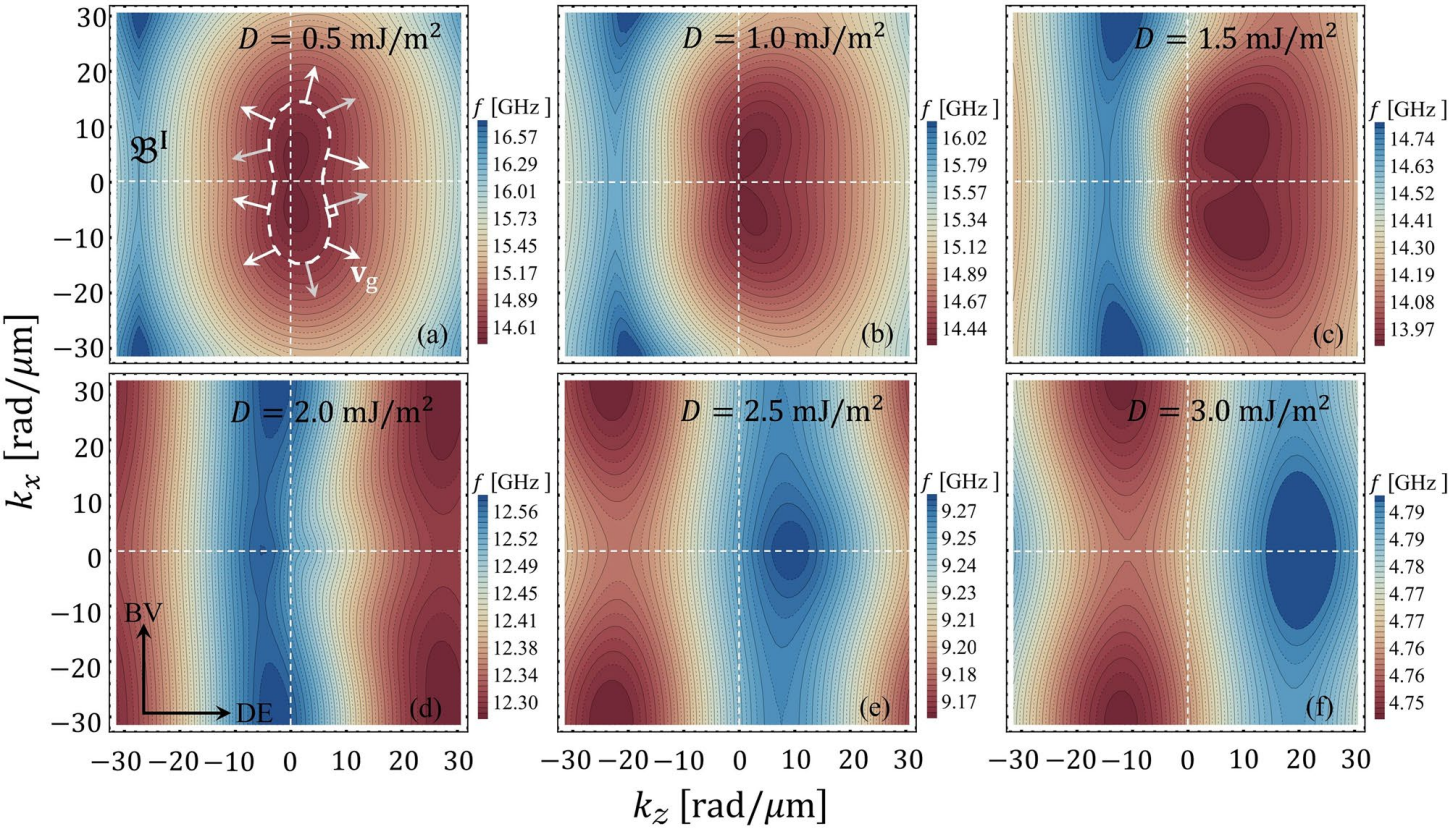
(**a**–**f**) Shows the isofrequency contours of band ${\mathfrak {B}}^{\mathrm{{I}}}$ projected in the plane of the wave-vector space for different values of *D*. In (**a**), the directions of the group velocity vector are illustrated along a given slowness surface.

Different values of *D* are considered, where one can see that $v^{z}_{\mathrm{g}}$ of the first band ${\mathfrak {B}}^{\mathrm{{I}}}$ tends to zero as the constant *D* increases. Similar behavior is noted on the band ${\mathfrak {B}}^{\mathrm{{III}}}$, but a different magnitude of *D* is required to reach the flat property. We can note that for some specific values of the DM constant, the group velocity becomes abruptly zero, which is related to the states where a maximum or minimum matches with the point ($\pi /a_z,0$). Moreover, the color change in some parts of Fig. 4b is correlated with the crossing between modes, which can happen at ($\pi /a_z,0$) for given values of *D*. In the case of the BV configuration, the group velocity $v^{x}_{\mathrm{g}}$ is about 3 orders of magnitude smaller than $v^{z}_{\mathrm{g}}$ (not shown), and $v^{x}_{\mathrm{g}}$ tends to reach magnitudes close to zero for lower values of the constant *D* as compared with DE modes.

**Figure 7:**
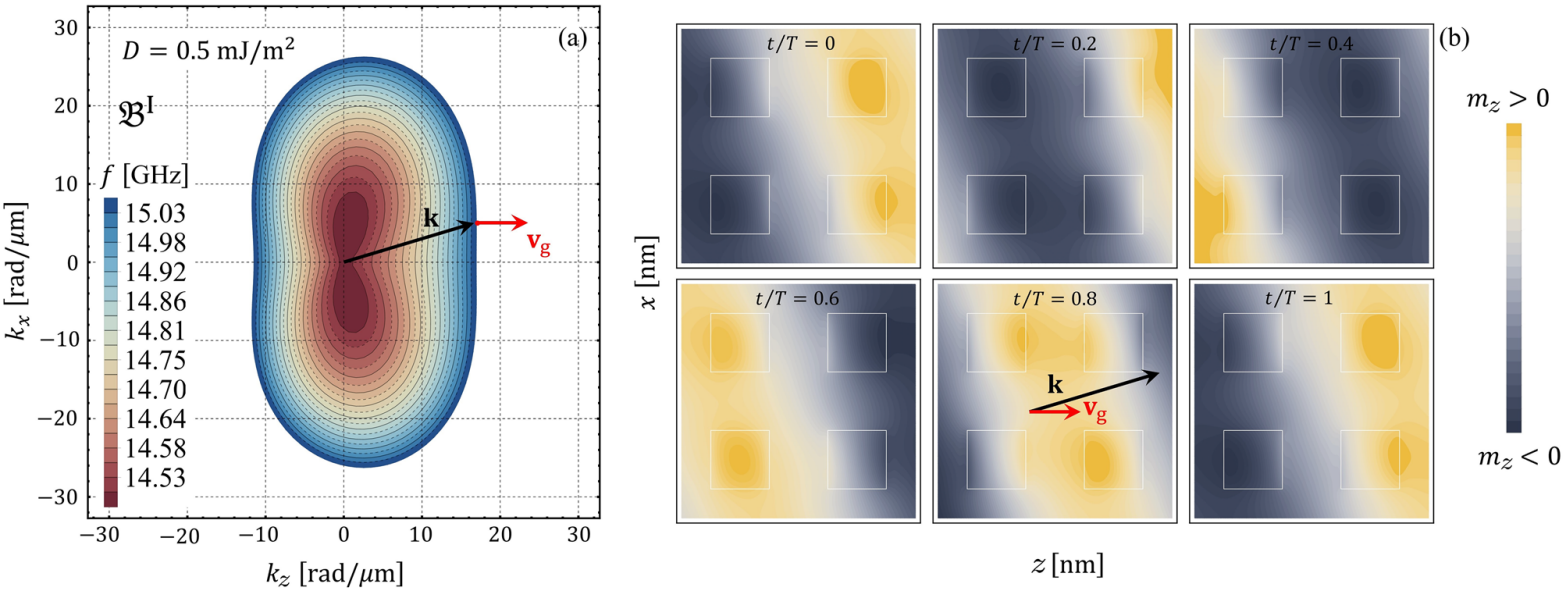
(**a**) Shows the isofrequency contour for the first band ${\mathfrak {B}}^{\mathrm{{I}}}$ evaluated at $D=0.5$ mJ/m$^{2}$. In (**b**), the spatio-temporal dependence of the *z*-component of the magnetization, $m_{z}$, is depicted. Here, $m_{z}$ is evaluated in arbitrary units and *T* is the period of the magnetization oscillations. The directions of the vectors ${\mathbf {k}}$ and ${\mathbf {v}}_{g}$ are also shown in (**b**).

The SW propagation for the DE and BV configurations is studied in the reduced Brillouin zone, where the magnitude of the DMI constant *D* is first varied while keeping the geometric parameters of the MC fixed. Another phenomenon worth analyzing is observing how the band structure is modified while altering the geometric parameters. In Fig. 5, the DM constant is kept fixed ($D=3$ mJ/m$^{2}$), while the lateral dimensions of the heavy-metal square islands are varied. Figure 5a depicts square islands with $w_{x}=w_{z}$ in the range 10 − 95 nm, where the period is kept fixed at $a_{x}=a_{z}=100$ nm. In Fig. 5a, the minimum and maximum frequencies of the bands (${\mathfrak {B}}_{\mathrm{min}}^{\mathrm{{n}}}$ and ${\mathfrak {B}}_{\mathrm{max}}^{\mathrm{{n}}}$) are calculated, for the first four bands, as a function of the filling fraction ($w_{x}w_{z}/a_{x}a_{z}$), which represents the portion of the ferromagnetic film covered with the heavy metal. The filling fraction plays an essential role in creating flat bands and bandgap widths. For the first band ${\mathfrak {B}}^{\mathrm{{I}}}$, from $w_{z,x}=30$ nm to 80 nm (or filling fraction from 0.09 to 0.64) the bands become flat. The third band ${\mathfrak {B}}^{\,\mathrm {III}}$ become flat from approximately $w_{z,x}=50$ nm to 80 nm (filling fraction between 0.25 and 0.64). For the bands ${\mathfrak {B}}^{\mathrm{{II}}}$ and ${\mathfrak {B}}^{\mathrm{{IV}}}$, there is no flat band observed for $D=3$ mJ/m$^{2}$. On the other side, the bandgap widths (defined by the difference ${\mathfrak {B}}^{\mathrm{{n+1}}}_{\mathrm{min}}-{\mathfrak {B}}^{\mathrm{{n}}}_{\mathrm{max}}$) are shown in Fig. 5b as function of the filling fraction and the square width (upper red scale). One can also note that the first and third bandgap widths reach a significant maximum compared to the second BG width. This behavior also occurs for low values of *D* but with reduced bandgap widths. In addition, the maximum of the first and second BG widths is reached at similar values of the island widths ($w_{z,x}$), while the third BG width occurs at a slightly larger $w_{z,x}$.

**Figure 8:**
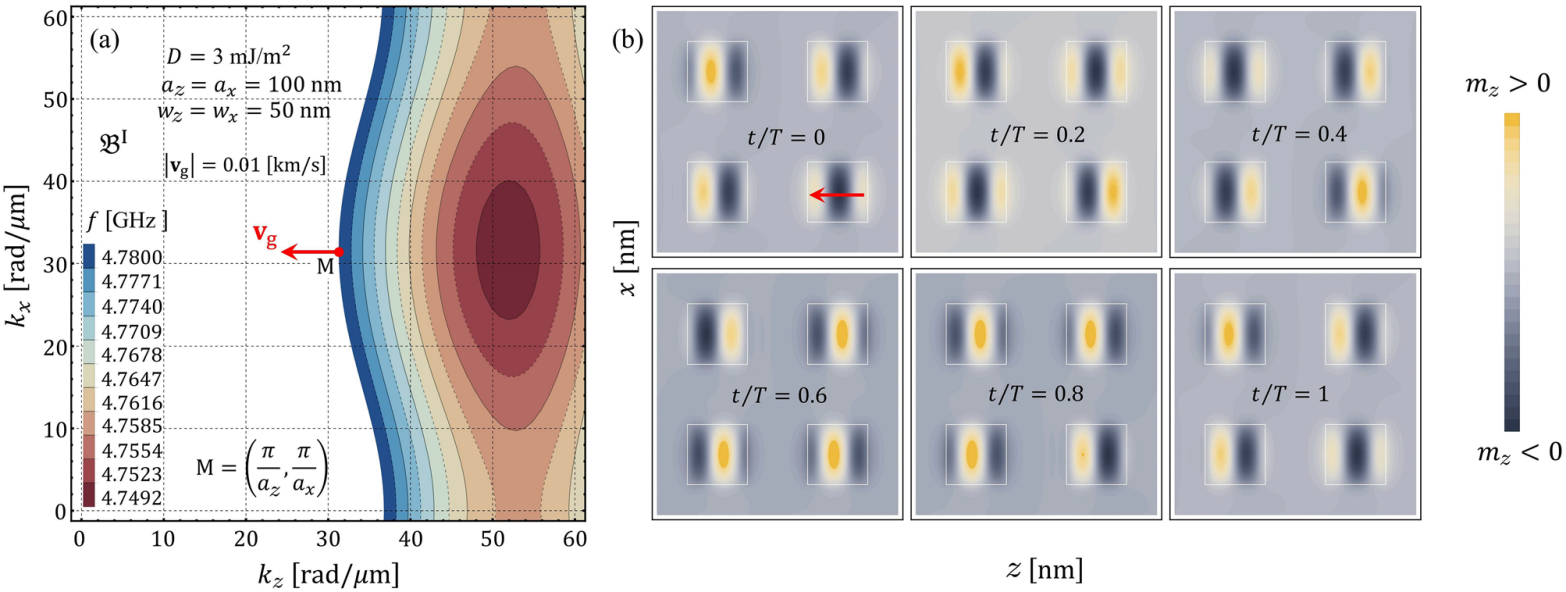
In (**a**), the isofrequency contour for the first band ${\mathfrak {B}}^{\mathrm{{I}}}$, evaluated at $D=3$ mJ/m$^{2}$, is depicted. In (**b**), the spatio-temporal profiles of the magnetization component $m_{z}$ are illustrated. Such a component is evaluated at point M of the reduced Brillouin zone and calculated in arbitrary units.

The isofrequency contours of the magnonic dispersion are explored to understand the SW propagation in the two-dimensional chiral magnonic crystal. Such contours, also named slowness surfaces, are curves of constant frequency drawn in wave vector space that provides information concerning the wave’s energy flux^24^. Thus, the isofrequency contours indicate the relative direction of the energy flow concerning the wave vector. For instance, a circular contour is obtained under the only presence of exchange interaction, which means that the wave propagates isotropically in preferred directions^84^. In contrast, the BV and DE behave differently under the dipolar interaction, channeling the wave’s energy in privileged directions^85^. In the case of a chiral interaction like an anisotropic exchange (DMI), or interlayer dynamic dipolar coupling, the wave’s energy can be channeled in only one direction due to the nonreciprocity induced by the chirality^84,85^. In Fig. 6a–f, the slowness surfaces are calculated as a function of the wave vector components for different values of *D*, where the color scale gives the constant frequency of each contour. In these graphs, it is again evident that the increased interfacial DMI induces the phenomenon of nonreciprocity of the SW propagation. Overall, as shown by Fig. 6a–c, nonreciprocal interference patterns can be designed (see details in Refs.^84,85^). In the particular case shown in Fig. 6c, the pronounced bump formed at low frequencies moves toward a larger $k_{z}$ as $D$. Besides, there are some directions where the curvature change in sign, which means there will be a zero curvature point so that caustic spin waves will arise. Note that the frequency difference between the maximum and the minimum of the band is less or equal to 0.1 GHz in the cases depicted in Fig. 6e,f. Hence, in such cases, the bands can be considered flat. The temporal evolution of the spin waves in real space is also studied. Figure 7a illustrates the isofrequency curves and Fig. 7b the in-plane dynamic magnetization component $m_{z}$ evaluated at different times, both for small DMI ($D=0.5$ mJ/m$^{2}$). By considering that the direction of the group velocity will be in the direction perpendicular to the tangent plane that is formed at a specific point of the isofrequency curve (see red arrow in Fig. 7a), we compare this direction with the time evolution of the in-plane component $m_{z}$. We can note that the direction of the wavefront propagation is along the wave vector ${\mathbf {k}}$, which is parallel to the phase velocity $v_{\mathrm{p}}=\omega /{\mathbf {k}}$, while in the zone with active DMI, the temporal evolution of the wave seems to be different. When the magnitude of the constant *D* increases, it is evident that there is a particular temporal evolution of the SW underneath the heavy metals, as shown in Fig. 8, where a constant $D=3$ mJ/m$^{2}$ was used. In other words, the magnon population is strongly localized in the active zones with DMI, while there are practically no excitations in the outer regions and the wavefront parallel to the phase velocity is not visible. Such a temporal behavior of the flat bands is consistent with the case of a one-dimensional chiral magnonic crystal, where the dispersionless modes are strongly localized in the zones in contact with heavy metal stripes^28,29^. Therefore, a two-dimensional periodic DM coupling can also reach a nontrivial time dependence of the magnetization for the flat bands in the proposed 2D magnonic architecture.

## Conclusion

The spin-wave spectra of two-dimensional chiral magnonic crystals are studied, consisting of a configuration of heavy-metal square islands in contact with an ultrathin ferromagnetic film. As the interfacial Dzyaloshinskii–Moriya constant increases, the frequency of the modes reduces, and indirect bandgaps are observed, which are caused by the nonreciprocity induced by the coupling. As the modes decrease in frequency, they are more prone to forming flat bands. For the two-dimensional magnonic crystal, an omnidirectional flattening of the modes is obtained so that in all directions, the low-frequency bands are dispersionless for significant values of the DM constant. In the first and third bands, the group velocity is considerably reduced when increasing the DM constant, which correlates with the flat character of the modes. The role of the filling fraction is also discussed, finding specific ranges for which the flat bands are likely to be observed. The examination of the isofrequency curves and the localization for the flat modes encountered a nontrivial temporal evolution of the magnetization and predicted a channelized propagation of the SWs. Part of the results has been compared with micromagnetic simulations, where a good agreement was reached between both methods. The two-dimensional chiral magnonic crystals harbor more interesting physical properties than other metamaterials since they exhibit a more controllable way of tuning omnidirectional flat bands and nonreciprocal magnonic propagation. These results open up new possibilities for realizing spin-wave-based logic devices.

## Data Availability

The datasets used and/or analysed during the current study are available from the corresponding author on reasonable request.
